# 3D AFM Nanomechanical Characterization of Biological Materials

**DOI:** 10.3390/nano13030395

**Published:** 2023-01-18

**Authors:** Stylianos Vasileios Kontomaris, Andreas Stylianou, Anastasios Georgakopoulos, Anna Malamou

**Affiliations:** 1 BioNanoTec Ltd., 2043 Nicosia, Cyprus; 2 Faculty of Engineering and Architecture, Metropolitan College, 15125 Athens, Greece; 3 School of Sciences, European University Cyprus, 2404 Nicosia, Cyprus; 4 School of Electrical and Computer Engineering, National Technical University of Athens, 15780 Athens, Greece

**Keywords:** average young’s modulus, nanoindentation, scanning probe microscopy, mechanical properties, depth-dependent behavior, cells, cancer diagnosis

## Abstract

Atomic Force Microscopy (AFM) is a powerful tool enabling the mechanical characterization of biological materials at the nanoscale. Since biological materials are highly heterogeneous, their mechanical characterization is still considered to be a challenging procedure. In this paper, a new approach that leads to a 3-dimensional (3D) nanomechanical characterization is presented based on the average Young’s modulus and the AFM indentation method. The proposed method can contribute to the clarification of the variability of the mechanical properties of biological samples in the 3-dimensional space (variability at the x–y plane and depth-dependent behavior). The method was applied to agarose gels, fibroblasts, and breast cancer cells. Moreover, new mathematical methods towards a quantitative mechanical characterization are also proposed. The presented approach is a step forward to a more accurate and complete characterization of biological materials and could contribute to an accurate user-independent diagnosis of various diseases such as cancer in the future.

## 1. Introduction

Atomic Force Microscopy (AFM) is a powerful instrument that offers the possibility of retrieving the mechanical properties of tissues, cells, fibrous components, and biomolecules [1,2,3,4,5,6,7,8,9,10]. It is important to note that the determination of the mechanical properties of biological materials at the nanoscale using the AFM indentation method has opened up new prospects regarding various biomedical applications such as disease diagnosis and prognosis (e.g., cancers and osteoarthritis) [1,4,5,6,7,8]. The major advantage of the AFM regarding the characterization of biological samples is that it can be applied in the characterization of single molecules and proteins as well as the characterization of complex biological samples such as cells and tissues [11].

However, the characterization of biological materials using AFM is still considered a challenging procedure. Many errors may arise which, among other factors [11], are related to data processing and the misuse of contact mechanics models that consider the sample to be an elastic half-space (biological samples at the nanoscale are highly heterogeneous and non-isotropic materials) [11,12]. The major problem regarding the lack of appropriate models and mathematical theories regarding AFM data processing has been very recently pinpointed [11]. The application of novel techniques for the mechanical characterization of biological samples requires the development of new theories and mathematical models for data processing. In this direction, several attempts have been recently proposed. In particular, a novel mathematical model for describing the depth-dependent mechanical properties of cells (that takes into account the surface tension effects) was recently derived by Ding et al. [13]. The mechanical properties of soft heterogeneous materials at the nanoscale can be also determined using the Trimechanic theory for general elastic response [14]. In this case, the applied force can be decomposed into three factors: the depth impact, the Hookean, and the tip-shape factor [14]. In addition, the depth-dependent nanomechanical properties of soft materials can be also determined using the average Young’s modulus theory [12,15].

It is important to note that the only way to achieve the accurate mechanical characterization of biological samples is to separately characterize the different superficial and inner nano-features in each case. For example, a simple model used to characterize the different mechanical properties of a cell’s membrane compared to its inner features has been proposed [16]. In addition, a recently published paper combined 3D acoustic manipulation with micro-indentation for 3-dimensional mechanical characterization at the microscale [17]. The need for an accurate 3D mechanical characterization using various approaches is growing fast, as was shown in [18], and is considered to be a cutting-edge area of research since the aim is to solve the most important problem in the mechanical characterization of biological samples, which is the current inability to record the mechanical properties at a 3D scale. In other words, existing AFM methods using typical Young’s modulus maps [19,20,21,22,23,24] provide a 2D characterization (since they do not take into account the alterations in mechanical properties’ in the 3rd dimension, i.e., as the indentation depth increases, and their results are user dependent).

In this paper, the previously developed ‘average Young’s modulus theory’ [12,15] is used for a complete mechanical characterization of biological materials at the nanoscale. The primary goal of this paper was to use the average Young’s modulus theory for a 3D mechanical nano-characterization of any soft biological sample. The previously developed mathematical technique for creating depth-dependent average Young’s modulus maps [15] is combined with classic methods (i.e., conventional Young’s modulus maps) to create 3D plots that present the mechanical properties of 3-dimensional regions. These plots will be a sum of M average Young’s modulus maps (for each value of indentation depth, a different map will be available). Thus, assuming that each average Young’s modulus map consists of N values, the proposed method will result in measurements over a 3–dimensional region. In addition, the average Young’s modulus distributions for each map are also obtained. Thus, the average Young’s modulus distributions with respect to the indentation depth plots are also available. The significance of these plots is that they allow for the easy monitoring of the ‘softening/stiffening behavior’ as the indentation depth increases (as in 3D maps) but also permit the testing of whether the sample tends towards a ‘homogeneous’ behavior or the heterogeneity increases for big indentation depths. The 3D mechanical characterization was applied to different types of cells, since these samples are of utmost scientific interest related to early cancer diagnosis [4] and other applications [25]. The accurate 3D mechanical characterization of biological samples will lead to a new strategy regarding AFM data processing and will open up new possibilities for the AFM use in research or industrial applications.

## 2. Materials and Methods

### 2.1. Depth Dependent Mechanical Properties

The procedure for determining the depth-dependent mechanical properties of biological samples has been previously presented [15]. Briefly, a heterogeneous material can be considered as the sum of N narrow homogeneous slices (each one of them has a thickness, Δ*h*). The average Young’s modulus of these slices is defined below:

(1) $$\bar{E}=\frac{E_{1}+E_{2}+\ldots +E_{N}}{N}=\frac{E_{1}\Delta h+E_{2}\Delta h+\ldots +E_{N}\Delta h}{N\Delta h}=\frac{1}{h}\sum_{i=1}^{N}E_{i}\Delta h\;$$

Assuming *N*→∞ layers with the same thickness, Equation (1) takes the following form:

(2) $$\bar{E}=\frac{1}{h}\int_{0}^{h}E\left(y\right)dy$$

The symbol $E\left(y\right)$ indicates the depth-dependent Young’s modulus function. The classic Hertzian equations for cylindrical, parabolic, or conical indenter are valid [15]; however, the parameter that is determined using a typical fitting procedure is the average Young’s modulus.

For cylindrical indenters,

(3) $$F=\frac{2\bar{E}R}{1-v^{2}}h$$

In Equation (3), R is the indenter’s radius, v is the Poisson’s ratio, F is the applied force on the sample, and h is the indentation depth. For conical indenters,

(4) $$F=\frac{2\left(tan\theta \right)}{\pi \left(1-v^{2}\right)}\bar{E}h^{2}$$

In Equation (4), θ is the cone’s half angle. For parabolic indenters (or spherical indenters for small indentation depths),

(5) $$F=\frac{4R^{1/2}\bar{E}}{3\left(1-v^{2}\right)}h^{3/2}$$

It is also significant to note that for spherical indenters, the h/R ratio is a significant parameter to consider. For example, the accurate equation that relates the applied force to the indentation depth for a spherical indentation on an elastic half space was derived by Sneddon [3] and is provided below:

(6) $$F=\frac{E}{2\left(1-v^{2}\right)}\left[\left(r_{c}^{2}+R^{2}\right)ln\left(\frac{R+r_{c}}{R-r_{c}}\right)-2r_{c}R\right]$$

In Equation (6), E is the Young’s modulus of the elastic half space. The indentation depth is related to the contact radius with the following equation [3]:

(7) $$ln\left(\frac{R+r_{c}}{R-r_{c}}\right)=\frac{2h}{r_{c}}$$

Nevertheless, an alternating equation was recently derived which directly correlates the applied force to the indentation depth [3]:

(8) $$F=\frac{4ER^{1/2}}{3\left(1-v^{2}\right)}h^{3/2}Z$$

The correction factor Z is provided below:

(9) $$Z=c_{1}+\frac{3}{4}c_{2}R^{-1/2}h^{1/2}+\frac{3}{6}c_{3}R^{-3/2}h^{3/2}+\frac{3}{8}c_{4}R^{-5/2}h^{5/2}+\ldots +\frac{3}{2N}c_{N}R^{\left(\frac{3}{2}-N\right)}h^{N-3/2}$$

or,

(10) $$Z=c_{1}+\sum_{M=2}^{N}\frac{3}{2Μ}c_{M}R^{\left(\frac{3}{2}-M\right)}h^{M-3/2},\;\;\;\;\;\;\;\;\;\;\;\;Z\leq 1$$

**Figure 1:**
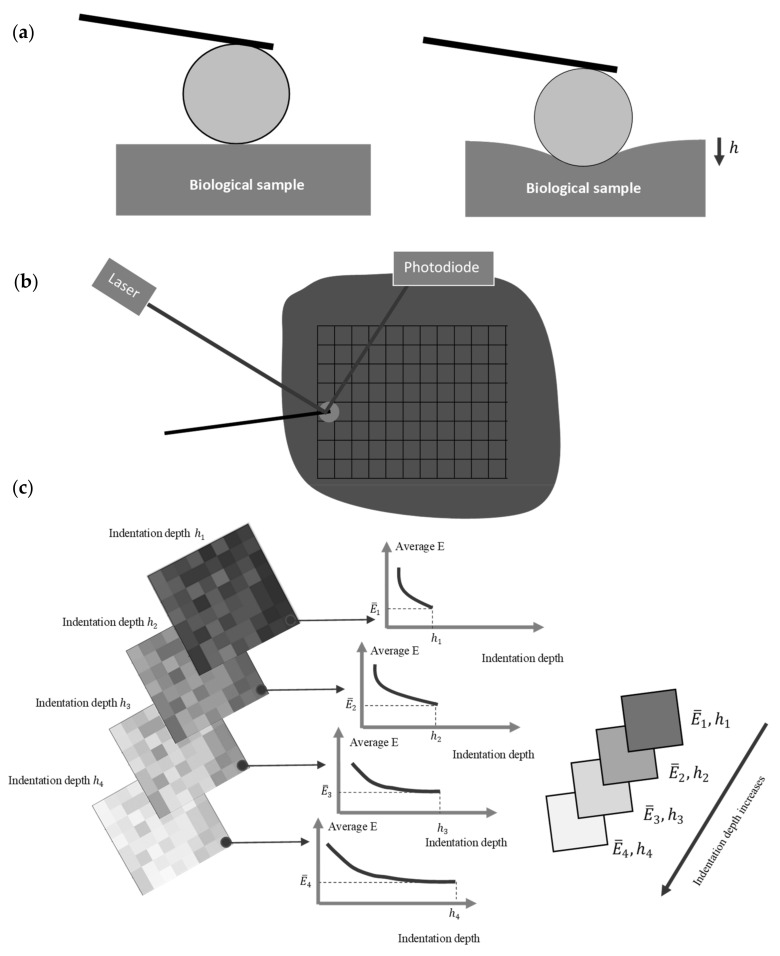
Methodology for the depth-dependent mechanical characterization of biological samples: (**a**) a force-indentation curve using a spherical indenter is obtained. (**b**) Multiple force–indentation curves are taken within an area of interest using AFM. (**c**) The average Young’s modulus is calculated for different indentation depths. Many average Young’s modulus maps are obtained (four representative maps are shown). Each map is valid for a specific indentation depth. In this figure, it is assumed that the average Young’s modulus decreases as the indentation depth increases.

To determine the depth-dependent mechanical properties at a specific point, a force–indentation depth curve is obtained. Biological samples are depth dependent; thus, for each value of indentation depth the average Young’s modulus will be different. Hence, many different fittings should be obtained using Equations (3)–(5) depending on the indenter’s shape. For an indentation depth equal to $h_{1}$ the average Young’s modulus will be equal to ${\bar{E}}_{1}$, and for an indentation depth equal to $h_{2}$, the average Young’s modulus will be equal to ${\bar{E}}_{2}$, etc. (Figure 1). Thus, the values ${\bar{E}}_{1}$, ${\bar{E}}_{2},\ldots {\bar{E}}_{N}$ for $0\leq h\leq h_{1}$, $0\leq h\leq h_{2}$, …,$0\leq h\leq h_{N}$ are obtained and the $\bar{E}=f\left(h\right)$ data is determined. For example, it has been previously shown that for cells, the appropriate function to describe the depth-dependent mechanical properties at a specific point is $\bar{E}=ah^{b}+c$, where a,b,c are fitting factors (a,c>0, b<0) [12].

### 2.2. 3D Maps

**Figure 2:**
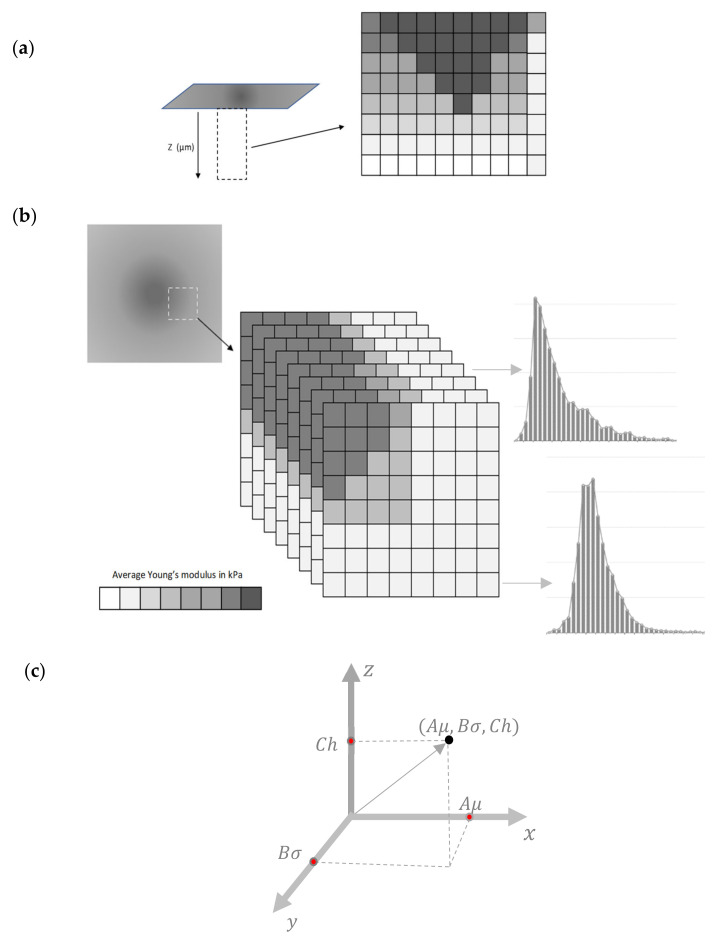
3D mechanical properties: (**a**) a depth dependent average Young’s modulus map [15]. The dark areas in the 3D plot presents nanoregions with big average Young’s modulus. The light color areas show nanoregions with small average E. (**b**) Eight average E maps and two representative average Young’s modulus distributions. (**c**) An arbitrary $\left(\mu ,\;\sigma ,\;h\right)$ vector.

At each region, a force–indentation depth curve will be obtained. Using these curves, the average Young’s modulus–indentation depth data will be obtained for each elementary region (Figure 2a). By deriving the $\bar{E}=f\left(h\right)$ data for many nanoregions, a big number of average Young’s modulus maps can be constructed (assume M the number of maps). The sum of M maps can then be used to derive a 3D plot, as presented in Figure 2b. The number of M maps that can be obtained depends on the accuracy that is needed to be acquired each time. For example, a big M number indicates an extremely detailed monitoring of the mechanical properties with respect to the indentation depth. At this point, it should be mentioned that the 3D maps are obtained using one experiment each time (since the $\bar{E}=f\left(h\right)$ data are derived using the conventional force–indentation data [12]). In other words, the experimental procedure used to yield a classic Young’s modulus map and the 3D map is the same. The difference is based on data processing. This is a major advantage of this method; the 3D mechanical characterization can be achieved without increasing the cost, the duration, or the complexity of the experimental procedure.

The 3D mechanical properties can be mathematically described using the average Young’s modulus distributions for each indentation depth. More specifically, the graph ‘probability with respect to the average elastic modulus’ for the sample can be plotted. The probability provides the occurrences of a single average Young’s modulus expressed as a fraction of 100. In Figure 2b, a characteristic illustration of a 3D plot consisting of eight average Young’s modulus maps is presented. In addition, two representative average Young’s modulus distributions are also shown. Each average Young’s modulus distribution can then fitted to an appropriate function. For example, assume that the average Young’s modulus distribution follows a Gaussian distribution for a specific indentation depth, $h_{1}$ (i.e., $0\leq h\leq h_{1}$):

(11) $$f\left(\bar{E}\right)=\frac{1}{\sigma \sqrt{2\pi }}e^{-\frac{1}{2}{\left(\frac{\bar{E}-\mu }{\sigma }\right)}^{2}}$$

In Equation (11), μ is the mean of the distribution and σ is the standard deviation. A mathematical criterion to evaluate the depth-dependent mechanical properties using elementary functions is to determine the $f\left(\bar{E}\right)$ function for each map. Using this approach, the μ,σ values for each map are also determined. More specifically,

$$\mathrm{Map}\;1:\;0\leq h\leq h_{1},\;\mu =\mu_{1},\;\sigma =\sigma_{1}\;\mathrm{Map}\;2:\;0\leq h\leq h_{2},\;\mu =\mu_{2},\;\sigma =\sigma_{2}\;\vdots \;\mathrm{Map}\;Μ:\;0\leq h\leq h_{Μ},\;\mu =\mu_{Μ},\;\sigma =\sigma_{Μ}$$

Assume, for example, that, $\mu_{1}$>$\mu_{2}>\ldots >\mu_{M}$ and $\sigma_{1}$>$\sigma_{2}>\ldots >\sigma_{M}$. This result indicates that the mean of the distribution reduces as the indentation depth increases (i.e., this is a sample’s softening behavior) and the standard deviation also reduces (the sample tends towards a ‘homogeneous’ behavior).

A characteristic example of a sample tending towards a ‘homogeneous behavior’ is presented in [12]. The multiple average Young’s modulus distributions (for every indentation depth) can be also described using the following function:

(12) $$f\left(\bar{E}\right)=c_{1}\frac{1}{\sigma_{1}\sqrt{2\pi }}e^{-\frac{1}{2}{\left(\frac{\bar{E}-\mu_{1}}{\sigma_{1}}\right)}^{2}}+c_{2}\frac{1}{\sigma_{2}\sqrt{2\pi }}e^{-\frac{1}{2}{\left(\frac{\bar{E}-\mu_{2}}{\sigma_{2}}\right)}^{2}}+c_{3}\frac{1}{\sigma_{3}\sqrt{2\pi }}e^{-\frac{1}{2}{\left(\frac{\bar{E}-\mu_{3}}{\sigma_{3}}\right)}^{2}}+\ldots +c_{N}\frac{1}{\sigma_{N}\sqrt{2\pi }}e^{-\frac{1}{2}{\left(\frac{\bar{E}-\mu_{N}}{\sigma_{N}}\right)}^{2}}$$

where, $c_{1}=1$, $c_{2}=c_{3}=\ldots =c_{N}=0$ for $h=h_{1}$, $c_{2}=1$, $c_{1}=c_{3}=\ldots =c_{N}=0$ for $h=h_{2}$,…, $c_{N}=1$, $c_{1}=c_{2}=\ldots =c_{N-1}=0$ for $h=h_{N}$. More specifically, when referring to the map for $h=h_{1}$, only the first term is kept, and when referring to the map for $h=h_{2}$, only the second term is kept, etc. Using Equation (12), it is also easy to plot the distributions for each indentation depth comparatively.

### 2.3. The (μ, σ, h) Vector

Since each map is characterized by three variables (i.e., *μ*, *σ*, *h*), the procedure can be simplified by assuming a vector in a 3-d space (*x*, *y*, *z*) (Figure 2c). In particular,

(13) $$\vec{r}=x\vec{i}+y\vec{j}+z\vec{k}$$

where, x=Aμ (*A* = 1 ${\mathrm{kPa}}^{-1}$), y=Bσ (*Β* = 1 ${\mathrm{kPa}}^{-1}$), and z=Ch ($C=1\;{\mathrm{nm}}^{-1})$. For example, assuming that for an average Young’s modulus map, μ=20 kPa, σ=5 kPa, and h=300 nm,

(14) $$\vec{r}=20\vec{i}+5\vec{j}+300\vec{k}$$

In case of a homogeneous sample, σ=0 and $\bar{E}=constant$ for any indentation depth. Thus, assuming $\bar{E}=20\;\mathrm{kPa}$, and for two different indentation depths (e.g., 100 nm and 300 nm),

(15) $${\vec{r}}_{1}=20\vec{i}+100\vec{k},\;{\vec{r}}_{2}=20\vec{i}+300\vec{k}$$

### 2.4. Software Development

**Figure 3:**
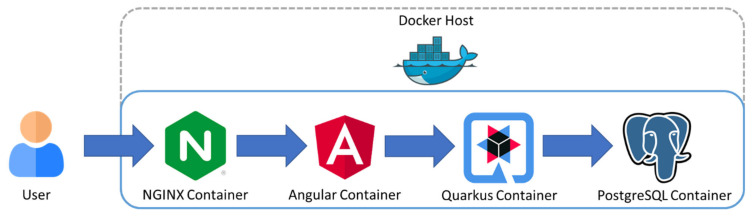
Software development: a web application appropriate to compute the average Young’s modulus at any point and visualize 3D mechanical properties maps and 3D distributions.

A web application was developed to compute the average Young’s modulus based on the mathematical modelling as described in previous sections and visualize the 3D mechanical properties maps and 3D distributions. This choice was driven by the need to be able to access the application by any device supporting modern web browsers. For the frontend, the Angular web framework was chosen using angular material and the state of the art web visualization library Apache Echarts. Additionally, for the backend, Quarkus java framework was used, with PostgreSQL for the database. An Nginx reverse proxy was configured so that all microservices can be accessed by the same address, with this creating the possibility to apply security measures in later application versions. The deployment was carried out using docker containers in order to be able to easily deploy the application to a cloud provider in the future. Figure 3 shows an overview of the application architecture that was implemented.

### 2.5. Agarose Gels

The measurements were performed using colloidal AFM probes (CP-PNPL-BSG-A, sQube, obtained by NanoAndMore GMBH, Wetzlar, Germany) with spheres of nominal radius equal to 1 μm. The indenters were firstly calibrated using the AFM test grating TGT1 (NT-MDT Instruments, Moscow, Russia). The experiments were conducted using agarose gels with a 2.5% concentration (low melting agarose, cat.no. CLSE-AG100, Cleaver Scientific, Warwickshire, United Kingdom) in a 35 mm petri dish. The Poisson’s ratio of an agarose gel can be assumed to be 0.5 due to the high water content.

### 2.6. Open Access Data

Open access nanoindentation data obtained using a conical indenter on a fibroblast was used [26]. According to this paper, human fibroblasts were cultured and maintained at 37 °C in a 5% CO_2_ humidified atmosphere. In this case, a conical indenter with a half angle equal to 25° was employed. The cantilever’s spring constant was 0.01 Nm^−1^ [26]. The (force–indentation) data used in this study were deposited in the (AtomicJ) repository (https://sourceforge.net/projects/jrobust/files/TestFiles/, accessed on 1 July 2022).

### 2.7. Experiments on Breast Cancer Cells

For the experiments, we used the 4T1 murine breast cells (4T1, ATCC). The cells were cultured in RPMI-1640 Medium and supplemented with 10% Foetal Bovine Serum (FBS) and with 1% antibiotic/antimycotic. The cells were cultured in a humidified incubator (culture incubator) at 37 °C with 95% O_2_ and 5% CO_2_ air conditions. AFM experiments were conducted after 24 h of culturing and the used cells were between the passages 8–10.

### 2.8. AFM Characterization

AFM measurements were performed in contact mode under liquid conditions in live cells and force curves were collected. A Molecular Imaging-Agilent PicoPlus AFM system was used. AFM characterization was obtained in liquid under complete medium [27] using 35 mm petri dishes. Force spectroscopy on live cells was performed with V-shaped soft silicon nitride probes (MLCT-Bio, cantilever C, Bruker) on an area of 5 × 5 μm^2^ at the center of each cell.

## 3. Results

### 3.1. An Approximately Homogeneous Sample

**Figure 4:**
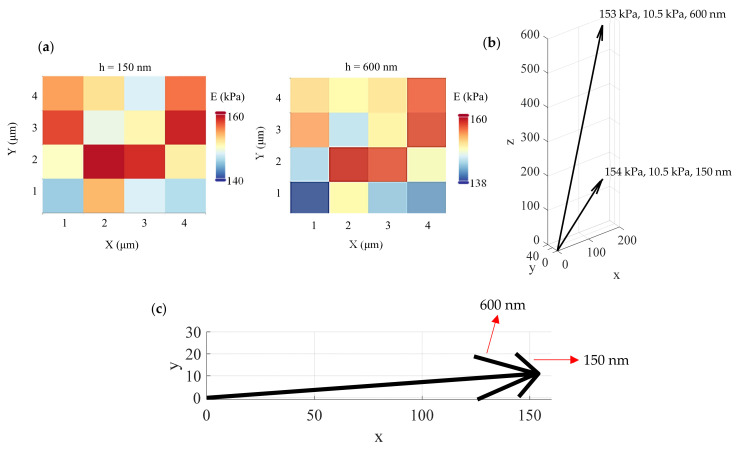
Experiments on agarose gel: (**a**) the proposed method can easily show that an agarose gel can be approximately considered as a homogeneous and isotropic material. (**b**) The *μ*, *σ* values are approximately the same for different indentation depths. (**c**) A 2D diagram showing the *μ*, *σ* values for indentation depths of 150 nm and 600 nm.

Firstly, agarose gels using the protocol described in Section 2.5 were tested. In Figure 4a, two average Young’s modulus maps were created, one for an indentation depth equal to 150 nm and one for an indentation depth equal to 600 nm. Each map consists of 16 measurements. The mean value of the average Young’s modulus for an indentation equal to 150 nm resulted in 154 kPa and a standard deviation equal to 10.5 kPa. For the second map (i.e., an indentation depth equal to 600 nm), the mean value resulted in 153 kPa and a standard deviation of 10.5 kPa. The results are similar with previous studies [28]. Thus, the proposed method can easily show that the agarose gel can be approximated to an elastic half space since the average Young’s modulus is depth-independent. The (*μ*, *σ*, *h*) vectors are also presented in Figure 4b,c.

### 3.2. Fibroblasts

**Figure 5:**
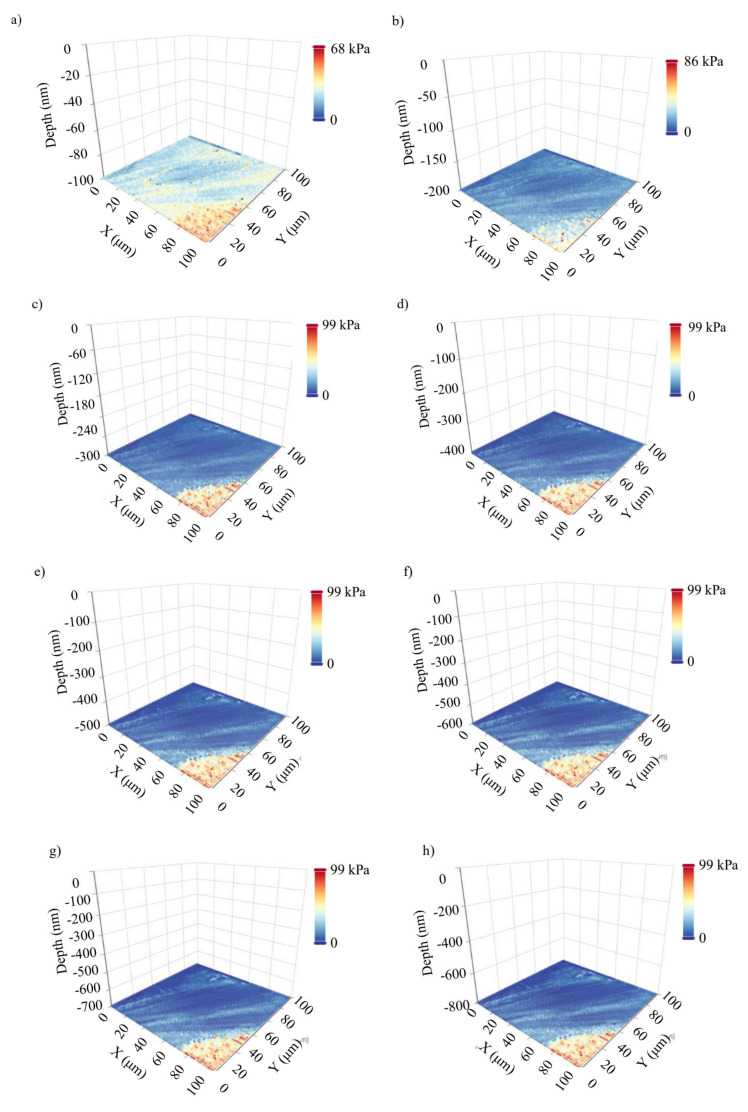
Experiments on fibroblasts: eight average Young’s modulus maps obtained on a fibroblast. The data were obtained from the AtomicJ repository (https://sourceforge.net/projects/jrobust/files/TestFiles/, accessed on 1 July 2022). The indentation depths for the presented maps were (**a**) 100 nm, (**b**) 200 nm, (**c**) 300 nm, (**d**) 400 nm, (**e**) 500 nm, (**f**) 600 nm, (**g**) 700 nm, and (**h**) 800 nm. The average Young’s modulus at each point changes as the indentation depth increases.

**Figure 6:**
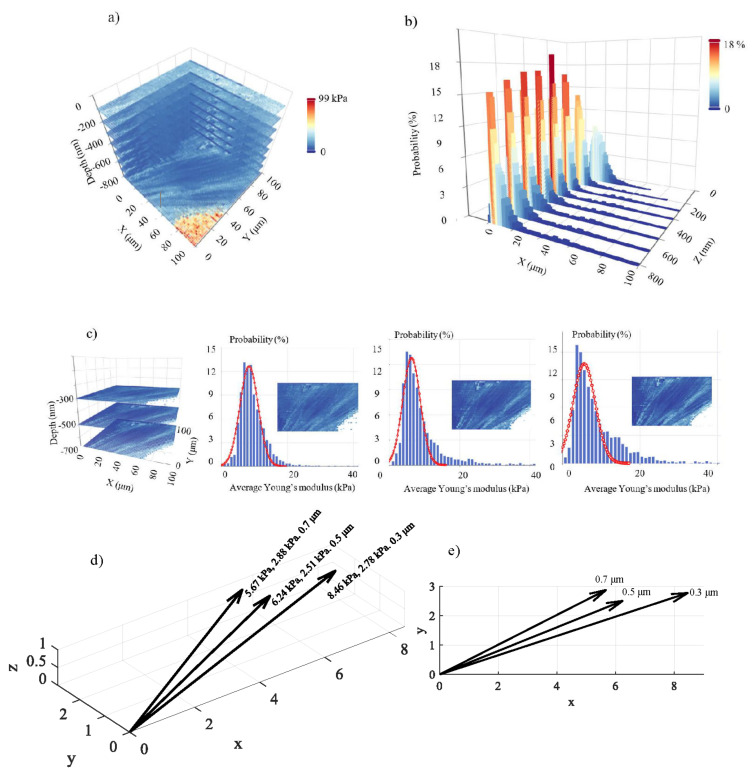
3D nanomechanical characterization: (**a**) a 3D mechanical properties map using eight average Young’s modulus maps on a fibroblast. The maximum indentation depth was 800 nm. (**b**) The average Young’s modulus distributions for the maps presented in Figure 5 and (**a**). (**c**) A 3D map using only three average Young’s modulus maps (300 nm, 500 nm, and 700 nm). The average Young’s modulus distributions are also presented. The data was fitted to Gaussian functions. (**d**) The (*μ*, *σ*, *h*) vectors. The data show a softening behavior as the indentation depth increases. (**e**) The (*μ*, *σ*, *h*) values in a 2D representation.

In Figure 5, 8 average Young’s modulus maps obtained on a fibroblast (open access data, see Section 2.6) are presented. The indentation depths were 100 nm, 200 nm, 300 nm, 400 nm, 500 nm, 600 nm, 700 nm, and 800 nm. Each map consists of 4096 average Young’s modulus values (64 × 64 measurements). The ‘yellow-red’ portion is affected by substrate effects. In Figure 6a, the 3D average Young’s modulus map using the eight maps presented in Figure 5 is shown. Additionally, the distribution of the average Young’s modulus for each map is presented in Figure 6b. In Figure 6c, a 3D map using only the data for 300 nm, 500 nm, and 700 nm is also presented. The portion of the data affected by the substrate effect has been removed, and the average Young’s modulus distributions are presented. The distribution was fitted to Gaussian functions (Equation (11)) for $h_{1}=300\;\mathrm{nm}$, $\mu_{1}=8.46\;\mathrm{kPa}$, and $\sigma_{1}=2.78\;\mathrm{kPa}$. In addition, for $h_{2}=500\;\mathrm{nm}$, $\mu_{2}=6.24\;\mathrm{kPa}$ and $\sigma_{2}=2.51\;\mathrm{kPa}$. Lastly, for $h_{3}=700\;\mathrm{nm}$, $\mu_{3}=5.67\;\mathrm{kPa}$ and $\sigma_{3}=2.88\;\mathrm{kPa}$. The (*μ*, *σ*, *h*) vectors for the three maps are also presented in Figure 6d,e. The fibroblast presents a ‘softening’ behavior as the indentation depth increases, as expected [12,13,29]. The standard deviation in every case is approximately the same.

### 3.3. Breast Cancer Cells

**Figure 7:**
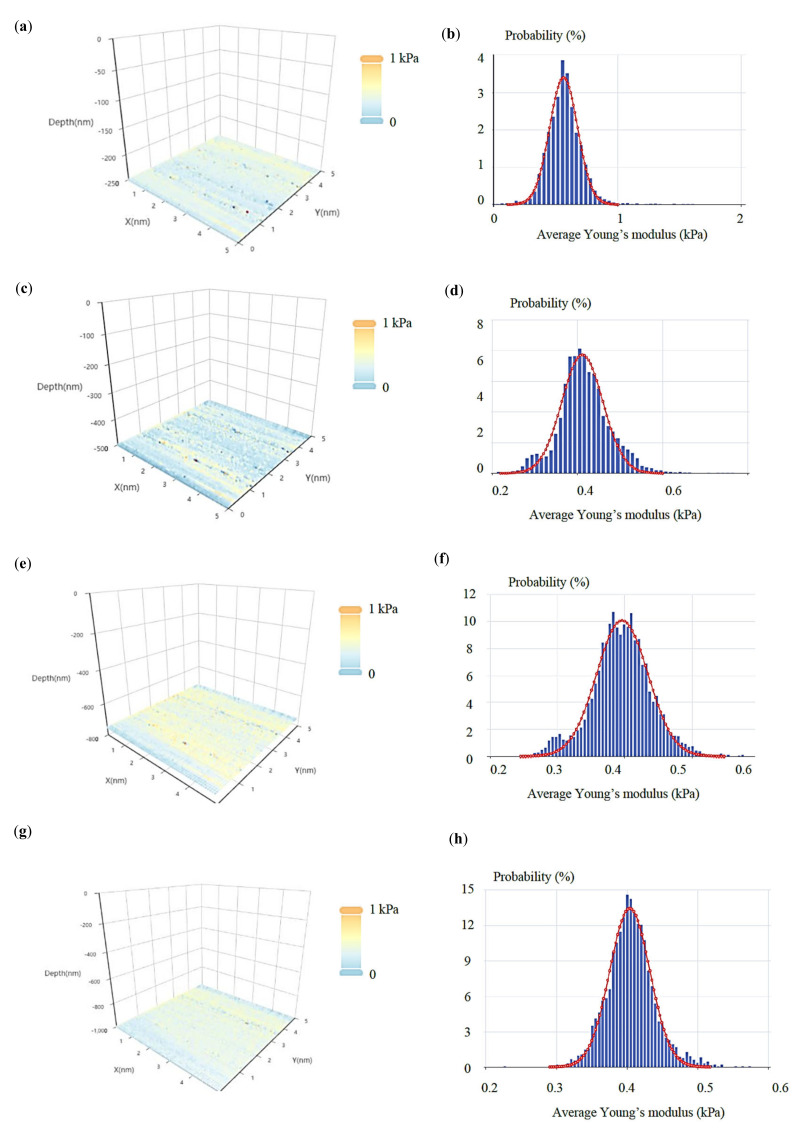
Experiments on breast cancer cells: four average Young’s modulus maps obtained on a breast cancer cell (see Section 2.7). For each map, the average Young’s modulus distribution is also presented. Each distribution was fitted to a Gaussian function (Equation (11)). The indentation depths for the presented maps and distributions were (**a**,**b**) 250 nm, (**c**,**d**) 500 nm, (**e**,**f**) 750 nm, and (**g**,**h**) 1000 nm.

**Figure 8:**
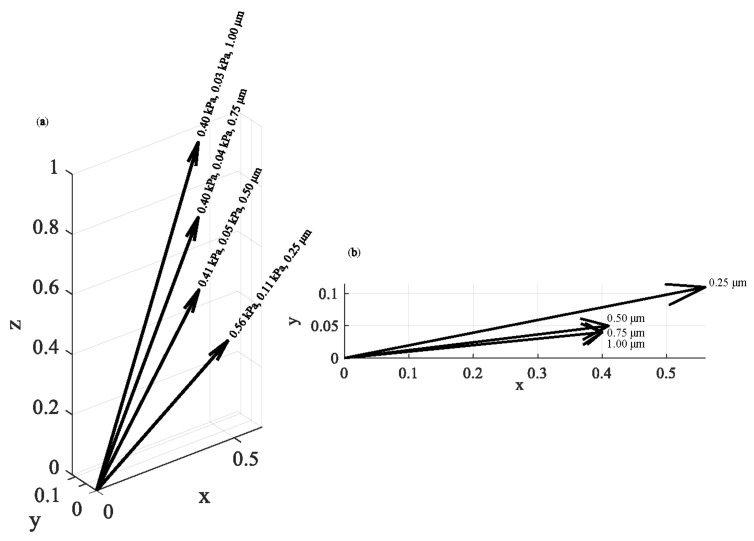
The (*μ*, *σ*, *h*) vectors: (**a**) the (*μ*, *σ*, *h*) vectors for experiments in breast cancer cells. The results show a ‘softening’ behavior as the indentation depth increases. (**b**) The (*μ*, *σ*, *h*) values in a 2D representation.

The same approach was applied on breast cancer cells (see protocol in Section 2.7). In Figure 7, four average Young’s modulus maps (with indentation depths of 250 nm, 500 nm, 750 nm, and 1000 nm) and the related average Young’s modulus distributions are presented. Each map consists of 64 × 64 = 4096 average Young’s modulus values. Each distribution was fitted to a Gaussian function. The (*μ*, *σ*, *h*) vectors were as follows: 0.56 kPa, 0.11 kPa, 250 nm, 0.41 kPa, 0.05 kPa, 500 nm, 0.40 kPa, 0.04 kPa, 750 nm, and 0.40 kPa, 0.03 kPa, 1000 nm. A ‘softening’ behavior is also recorded in this case. In addition, the standard deviation decreases as the indentation depth increases. In Figure 8, the (*μ*, *σ*, *h*) vectors are also presented.

## 4. Discussion

In this paper, a novel method for an accurate 3D nanomechanical characterization of biological materials was presented. The ability to determine the average Young’s modulus with respect to the indentation depth data enables the opportunity to develop 3D mechanical properties plots regarding biological samples. The method is based on obtaining multiple average Young’s modulus maps for different indentation depths. Conventional AFM techniques using classic Young’s modulus maps present a major limitation. They do not take into account the depth-dependent mechanical properties of highly heterogeneous materials such as biological ones. In other words, when using a conventional Young’s modulus map, the variation in terms of Young’s modulus is recorded over the x–y plane, but it is assumed that the calculated Young’s modulus is independent of the indentation depth. Thus, this paper goes beyond the state of the art by proposing a novel technique that do not present the aforementioned limitation. In the case of a fibroblast, 64 × 64 × 8 average Young’s modulus values presented a complete 3D characterization at the domain 0≤h≤800 nm (Figure 5 and Figure 6). In case of a breast cancer cell, 64 × 64 × 4 values were used to monitor the 3D mechanical properties at the domain 0≤h≤1000 nm (Figure 7). The exact number of measurements depends on the detail that needs to be obtained. In addition to the 3D maps, two other significant tools were also presented: the 3D mechanical distribution (i.e., multiple average Young’s modulus distributions depending on the indentation depth) and the (*μ*, *σ*, *h*) vectors. The 3D mechanical distribution (Figure 6b) is a significant tool since it shows in a quantitative way how the mechanical properties change with depth. The major advantage of this approach compared to average Young’s modulus maps is that it can be expressed in the form of a function. For example, the three distributions presented in Figure 6c can be expressed as follows:

(16) $$f\left(\bar{E}\right)=c_{1}\frac{1}{\sigma_{1}\sqrt{2\pi }}e^{-\frac{1}{2}{\left(\frac{\bar{E}-\mu_{1}}{\sigma_{1}}\right)}^{2}}+c_{2}\frac{1}{\sigma_{2}\sqrt{2\pi }}e^{-\frac{1}{2}{\left(\frac{\bar{E}-\mu_{2}}{\sigma_{2}}\right)}^{2}}+c_{3}\frac{1}{\sigma_{3}\sqrt{2\pi }}e^{-\frac{1}{2}{\left(\frac{\bar{E}-\mu_{3}}{\sigma_{3}}\right)}^{2}}$$

where, $c_{1}=1$, $c_{2}=c_{3}=0$ for h=300 nm, $c_{2}=1$, $c_{1}=c_{3}=0$ for h=500 nm, and $c_{3}=1$, $c_{1}=c_{2}=0$ for h=700 nm. In addition, $\mu_{1}=8.46\;\mathrm{kPa}$, $\sigma_{1}=2.78\;\mathrm{kPa}$, $\mu_{2}=6.24\;\mathrm{kPa}$, $\sigma_{2}=2.51\;\mathrm{kPa}$, $\mu_{3}=5.67\;\mathrm{kPa}$, and $\sigma_{3}=2.88\;\mathrm{kPa}$.

For the case of the breast cancer cells (Figure 7),

(17) $$f\left(\bar{E}\right)=c_{1}\frac{1}{\sigma_{1}\sqrt{2\pi }}e^{-\frac{1}{2}{\left(\frac{\bar{E}-\mu_{1}}{\sigma_{1}}\right)}^{2}}+c_{2}\frac{1}{\sigma_{2}\sqrt{2\pi }}e^{-\frac{1}{2}{\left(\frac{\bar{E}-\mu_{2}}{\sigma_{2}}\right)}^{2}}+c_{3}\frac{1}{\sigma_{3}\sqrt{2\pi }}e^{-\frac{1}{2}{\left(\frac{\bar{E}-\mu_{3}}{\sigma_{3}}\right)}^{2}}+c_{4}\frac{1}{\sigma_{4}\sqrt{2\pi }}e^{-\frac{1}{2}{\left(\frac{\bar{E}-\mu_{4}}{\sigma_{4}}\right)}^{2}}$$

where, $c_{1}=1$, $c_{2}=c_{3}=c_{4}=0$ for h=250 nm, $c_{2}=1$, $c_{1}=c_{3}=c_{4}=0$ for h=500 nm, …, $c_{3}=1$, $c_{1}=c_{2}=c_{4}=0$ for h=750 nm, and $c_{4}=1$, $c_{1}=c_{2}=c_{3}=0$ for h=1000 nm. In addition, $\mu_{1}=0.56\;\mathrm{kPa}$, $\sigma_{1}=0.11\;\mathrm{kPa}$, $\mu_{2}=0.41\;\mathrm{kPa}$, $\sigma_{2}=0.05\;\mathrm{kPa}$, $\mu_{3}=0.40\;\mathrm{kPa}$, $\sigma_{3}=0.04\;\mathrm{kPa}$, $\mu_{4}=0.40\;\mathrm{kPa}$, and $\sigma_{4}=0.03\;\mathrm{kPa}$. Finally, an easier method for determining the mechanical behavior of 3D areas is by using the (*μ*, *σ*, *h*) vectors (Figure 6d,e and Figure 8). Using this approach, it is easy to present how the mean value of the average Young’s modulus and the standard deviation changes as the indentation depth increases. For example, a ‘softening’ or a ‘stiffening’ behavior can be easily recorded. In addition, the possibility of a sample tending towards certain characteristics in a constant value as the indentation depth increases can also be monitored. In this case, the standard deviation should reduce as the indentation depth increases (i.e., the Gaussians should be ‘steeper’ for big indentation depths).

It is significant to note that 3D nanomechanical characterization may lead to easily reproducible results regarding cancer diagnosis. As it has been previously reported, cancer diagnosis can be performed using Young’s modulus distributions on normal/benign and cancer tissues [30]. AFM revealed that, in the case of normal/benign tissues, the stiffness distribution consists of one single peak, while in cancer tissues, at least two different peaks are determined due to the softening of the cancerous cells (Lower Elasticity Peak (LEP)) and the stiffening of the surrounding tissue (Higher Elasticity Peak (HEP)) [4,30]. However, to date, there is not a conclusive answer regarding the maximum indentation depth that should be used. Hence, multiple average Young’s modulus distributions for different indentation depths can be acquired through the use of 3D mechanical properties maps. Thus, the ‘two peak’ stiffness distribution on cancer tissues can be monitored as the indentation depth increases.

In addition, the possibility of monitoring the mechanical properties of a malignant tissue and its surroundings is important since local slight mechanical alterations could provide information regarding the metastasis procedure. A basic goal is to discover the paths used by cancer cells during metastasis based on the mechanical properties of the surroundings of the tumor. Thus, it will likely be possible to determine whether a metastasis procedure is in progress.

Another significant application for this method will be cancer prognosis. The accurate 3D monitoring of the mechanical properties of a malignant tissue under specific treatments is important to evaluate the effects of those treatments. Thus, 3D mechanical monitoring will likely become a powerful tool in medical doctors’ hands in decision making regarding the best personalized treatment procedure in the future.

## 5. Conclusions

In this paper, a 3D nanomechanical characterization based on the average Young’s modulus was presented. Using this approach, highly heterogeneous materials such as biological samples can be mechanically characterized in three dimensions. Other useful tools were also presented, such as the average Young’s modulus distributions with respect to indentation depth and the (*μ*, *σ*, *h*) vectors. In conclusion, this paper is a step forward towards the complete characterization of biological materials at the nanoscale.

## Data Availability

Data from AtomicJ software are referenced in the manuscript and a link is provided.

## References

[1] Stylianou A., Kontomaris S.V., Grant C., Alexandratou E. (2019). Atomic Force Microscopy on Biological Materials Related to Pathological Conditions. Scanning.

[2] Kontomaris S.V., Stylianou A., Yova D., Balogiannis G. (2015). The effects of UV irradiation on collagen D-band revealed by atomic force microscopy. Scanning.

[3] Kontomaris S.V., Malamou A., Stylianou A. (2022). The Hertzian theory in AFM nanoindentation experiments regarding biological samples: Overcoming limitations in data processing. Micron.

[4] Stylianou A., Lekka M., Stylianopoulos T. (2018). AFM assessing of nanomechanical fingerprints for cancer early diagnosis and classification: From single cell to tissue level. Nanoscale.

[5] Lekka M. (2012). Atomic force microscopy: A tip for diagnosing cancer. Nat. Nanotechnol..

[6] Stylianopoulos T., Munn L.L., Jain R.K. (2018). Reengineering the physical microenvironment of tumors to improve drug delivery and efficacy: From mathematical modeling to bench to bedside. Trends Cancer.

[7] Stolz M., Gottardi R., Raiteri R., Miot S., Martin I., Imer R., Staufer U., Raducanu A., Dueggelin M., Baschong W. (2009). Early detection of aging cartilage and osteoarthritis in mice and patient samples using atomic force microscopy. Nat. Nanotechnol..

[8] Stolz M., Raiteri R., Daniels A.U., Van Landingham A.M.W.R., Baschong W., Aebi U. (2004). Dynamic elastic modulus of porcine articular cartilage determined at two different levels of tissue organization by indentation-type atomic force microscopy. Biophys. J..

[9] Drolle E., Hane F., Lee B., Leonenko Z. (2014). Atomic force microscopy to study molecular mechanisms of amyloid fibril formation and toxicity in Alzheimer’s disease. Drug Metab. Rev..

[10] Li N., Jang H., Yuan M., Li W., Yun X., Lee J., Du Q., Nussinov R., Hou J., Lal R. (2017). Graphite-templated amyloid nanostructures formed by a potential pentapeptide inhibitor for Alzheimer’s disease: A combined study of real-time atomic force microscopy and molecular dynamics simulations. Langmuir.

[11] Krieg M., Fläschner G., Alsteens D., Gaub B.M., Roos W.H., Wuite G.J.L., Gaub H.E., Gerber C., Dufrêne Y.F., Müller D.J. (2019). Atomic force microscopy-based mechanobiology. Nat. Rev. Phys..

[12] Kontomaris S.V., Georgakopoulos A., Malamou A., Stylianou A. (2021). The average Young’s modulus as a physical quantity for describing the depth-dependent mechanical properties of cells. Mech. Mater..

[13] Ding Y., Wang J., Xu G.K., Wang G.F. (2018). Are elastic moduli of biological cells depth dependent or not? Another explanation using a contact mechanics model with surface tension. Soft Matter.

[14] Chen S.W., Teulon J.M., Kaur H., Godon C., Pellequer J.L. (2023). Nano-structural stiffness measure for soft biomaterials of heterogeneous elasticity. Nanoscale Horiz..

[15] Kontomaris S.V., Stylianou A., Georgakopoulos A., Malamou A. (2022). Is it mathematically correct to fit AFM data (obtained on biological materials) to equations arising from Hertzian mechanics?. Micron.

[16] Brill-Karniely Y. (2020). Mechanical Measurements of Cells Using AFM: 3D or 2D Physics?. Front. Bioeng. Biotechnol..

[17] Läubli N.F., Burri J.T., Marquard J., Vogler H., Mosca G., Vertti-Quintero N., Shamsudhin N., de Mello A., Grossniklaus U., Ahmed D. (2021). 3D mechanical characterization of single cells and small organisms using acoustic manipulation and force microscopy. Nat. Commun..

[18] Troyanova-Wood M.A., Coker Z., Bixler J., Ibey B. Brillouin imaging for noncontact and label-free 2D and 3D mapping of cellular mechanical properties. Proceedings of the Label-free Biomedical Imaging and Sensing (LBIS) 2021.

[19] Islam M.T., Tang S., Liverani C., Saha S., Tasciotti E., Righetti R. (2020). Non-invasive imaging of Young’s modulus and Poisson’s ratio in cancers in vivo. Sci. Rep..

[20] Chen X., Hughes R., Mullin N., Hawkins R.J., Holen I., Brown N.J., Hobbs J.K. (2020). Mechanical Heterogeneity in the Bone Microenvironment as Characterized by Atomic Force Microscopy. Biophys. J..

[21] Bontempi M., Salamanna F., Capozza R., Visani A., Fini M., Gambardella A. (2022). Nanomechanical Mapping of Hard Tissues by Atomic Force Microscopy: An Application to Cortical Bone. Materials.

[22] Achterberg V.F., Buscemi L., Diekmann H., Smith-Clerc J., Schwengler H., Meister J.J., Wenck H., Gallinat S., Hinz B. (2014). The nano-scale mechanical properties of the extracellular matrix regulate dermal fibroblast function. J. Investig. Dermatol..

[23] Asgari M., Latifi N., Giovanniello F., Espinosa H.D., Amabili M. (2022). Revealing Layer-Specific Ultrastructure and Nanomechanics of Fibrillar Collagen in Human Aorta via Atomic Force Microscopy Testing: Implications on Tissue Mechanics at Macroscopic. Scale Adv. NanoBiomed Res..

[24] Vaez M., Asgari M., Hirvonen L., Bakir G., Khattignavong E., Ezzo M., Aguayo S., Schuh C.M., Gough K., Bozec L. (2022). Modulation of the biophysical and biochemical properties of collagen by glycation for tissue engineering applications. Acta Biomater..

[25] Wu P.H., Aroush D.R.B., Asnacios A., Chen W.C., Dokukin M.E., Doss B.L., Durand-Smet P., Ekpenyong A., Guck J., Guz N.V. (2018). A comparison of methods to assess cell mechanical properties. Nat. Methods.

[26] Hermanowicz P., Sarna M., Burda K., Gabryś H. (2014). An open source software for analysis of force curves. Rev. Sci. Instrum..

[27] Sokolov I. (2007). Chapter 1: Atomic Force Microscopy in Cancer Cell Research, in Cancer Nanotechnology.

[28] Kontomaris S.V., Malamou A., Stylianou A. (2022). A New Approach for the AFM-Based Mechanical Characterization of Biological Samples. Scanning.

[29] Pogoda K., Jaczewska J., Wiltowska-Zuber J., Klymenko O., Zuber K., Fornal M., Lekka M. (2012). Depth-sensing analysis of cytoskeleton organization based on AFM data. Eur. Biophys. J..

[30] Plodinec M., Loparic M., Monnier C.A., Obermann E.C., Zanetti-Dallenbach R., Oertle P., Hyotyla J.T., Aebi U., Bentires-Alj M., Lim R.Y.H. (2012). The nanomechanical signature of breast cancer. Nat. Nanotech..

